# Prevention and Management of Postoperative Infection After Anterior Cruciate Ligament Reconstruction: A Narrative Review

**DOI:** 10.3390/jcm14020336

**Published:** 2025-01-08

**Authors:** Efstathios Konstantinou, Thomas Pfeiffer, Michael S. Rocca, Camila Grandberg, Karina Dias, Volker Musahl

**Affiliations:** 1 Department of Orthopaedic Surgery, UPMC Freddie Fu Sports Medicine Center, University of Pittsburgh Medical Center, 3200 S Water St., Pittsburgh, PA 15203, USA; efstathios.konstantinou@gmail.com (E.K.); grandbergc@upmc.edu (C.G.); diask@upmc.edu (K.D.); musahlv@upmc.edu (V.M.); 2 Cologne Merheim Medical Center, Witten/Herdecke University, 51109 Cologne, Germany; pfeiffert@kliniken-koeln.de; 3 Department of Experimental Sports Traumatology, Witten/Herdecke University, 58455 Witten, Germany

**Keywords:** anterior cruciate ligament, infection, prevention, grafts, biofilms, postoperative complications, arthroscopy

## Abstract

**Background**: Postoperative infection following anterior cruciate ligament reconstruction (ACLR) is a rare yet severe complication that can compromise patient outcomes, leading to prolonged recovery, graft failure, and knee dysfunction. Although infection rates are reported to be less than 2%, it remains essential to implement strategies to reduce infection risk and improve surgical outcomes. **Methods**: This review explores current evidence on the prevention of infections in ACLR, emphasizing the importance of timely antibiotic prophylaxis and vancomycin presoaking of grafts, which has been associated with a substantial reduction in infection rates. **Results**: Empirical antibiotic therapy should be started immediately after joint aspiration when infection is suspected. Treatment must prioritize culture-specific antibiotic regimens to optimize patient outcomes. Surgical intervention with arthroscopic debridement and irrigation needs to occur as soon as the diagnosis of infection is made. Often, this is performed with a focus on retaining the graft in order to preserve knee stability, if possible. Careful intraoperative management, along with the aid of infectious disease specialists, is paramount to help optimize outcomes following infection after ACLR. **Conclusions**: This review emphasizes the need for treatment protocols and highlights areas for future research to establish clear guidelines on infection after ACLR, especially with decisions of graft retention versus removal.

## 1. Introduction

Postoperative infection following anterior cruciate ligament reconstruction (ACLR) is a potentially catastrophic complication that results in prolonged recovery and poor functional outcomes [1]. While postoperative infection following ACLR is uncommon, with reported incidence rates from 0.1% to 2%, it is crucial to promptly diagnose infection to prevent severe complications such as knee dysfunction, cartilage damage, or arthrofibrosis [2,3]. Additionally, a large-scale retrospective study of pediatric and adolescent patients undergoing ACLR showed an overall infection rate of 0.52%, which is similar to that reported in adults [4]. Within this cohort, patients under the age of 15 had a lower infection rate, suggesting a potential age-related difference in infection susceptibility.

The majority of infections that occur after ACLR are acute postoperative infections [4]. If the postoperative infection after ACLR is not promptly recognized and treated, it may result in either acute or long-term knee dysfunction due to cartilage damage or failure of the ACL graft [5]. Management of infection after ACLR is challenging, and surgeons must decide to either retain or remove the ACL graft at times of debridement. When appropriate, retaining the ACL graft may be crucial in preserving knee stability and function, reducing the need for complex revision procedures, and improving long-term outcomes [6]. Furthermore, individuals who experience infection after ACLR are at high risk for undergoing multiple procedures and experiencing inferior functional recovery. Patients experiencing postoperative ACLR infection also have an increased likelihood of hospital readmission [6,7]. As a result, patient quality of life and ability to return to work or participate in sports may be significantly affected [8].

The prevention of infections following ACLR is crucial and involves multiple factors, including preoperative patient preparation, aseptic surgical techniques, and the use of prophylactic antibiotics. An emerging area of focus in infection prevention is the management of biofilms, which are known to contribute to persistent infections. Understanding the biofilm’s role in graft infections and the biofilm’s burden on the components or materials used for graft fixation may have the potential to help guide strategies for improved prevention and control. Preoperative preparation includes optimizing patient health, ensuring proper skin disinfection, and using appropriate hair removal techniques to minimize infection risk. Additionally, structured prevention programs, such as standardized antibiotic protocols, improved sterile techniques, and enhanced postoperative care, have effectively reduced infection rates in ACLR and other orthopedic procedures [9]. This review aims to provide a comprehensive overview of preventive measures, differences in ACL grafts, causative pathogens for infection, and the various management approaches for infection following ACLR. The objective of this review is to investigate methods for reducing infection rates in ACLR and approaches for effectively managing the challenging complication of infection after ACLR.

## 2. Methods

This review was conducted to examine and compile evidence on the prevention and management of infections following ACLR. A comprehensive search was performed using the MEDLINE, PubMed, and Google Scholar databases, covering studies published in English prior to September 2024. The search strategy was focused on ACLR and infection, as well as treatment strategies and prevention. The search terms included combinations of keywords such as ‘anterior cruciate ligament’, ‘infection’, ‘prevention’, ‘grafts’, ‘biofilms’, ‘postoperative complications’, and ‘arthroscopy’. The database search was conducted by E.K., T.P., K.D., C.G., and M.S.R., who independently reviewed the results of the search to ensure the inclusion of relevant studies.

This review focused on key aspects such as infection prevention measures, the role of graft types, common pathogens, and approaches to infection management. Studies were included based on their relevance to the topic, regardless of study design. This approach allowed for the integration of evidence from prospective and retrospective studies, meta-analyses, systematic reviews, and case reports. The findings were critically analyzed and synthesized to provide a comprehensive understanding of the topic. A summary of the key findings is presented in Table 1.

## 3. Skin Preparation

Skin preparation and wound management are critical components in reducing the risk of infections in ACLR. Hair removal from the surgical incision site ensures hair does not interfere with incision exposure, wound closure, or wound dressing while reducing the risk of surgical site infections (SSIs) by eliminating potential bacterial contamination [10,11,12]. The method and timing of hair removal significantly influence SSI risk. Evidence suggests that hair clippers or depilatory creams are preferable to razors, as razors may cause skin abrasions leading to pseudofolliculitis and SSIs [11,12,13]. Shaving on the day of surgery slightly reduces SSI incidence compared to shaving the day before, emphasizing the importance of timing [14]. Current guidelines from the Centers for Disease Control and Prevention (CDC) and the Association of periOperative Registered Nurses (AORN) recommend removing hair as close to surgery as possible and outside the operating room [13,15,16]. However, not removing hair may have a minimal difference in SSI risk compared to using clippers or creams, leaving the decision to the surgeon’s discretion due to insufficient evidence for consensus [11].

Proper skin preparation is critical for achieving a sterile operative environment. Common antiseptics include chlorhexidine, isopropyl alcohol, and povidone-iodine [17]. A meta-analysis found chlorhexidine more effective than povidone-iodine in reducing overall SSI rates, though the study’s heterogeneity limits its applicability to arthroscopic or orthopedic procedures [18]. In lower-limb trauma surgery, povidone-iodine–alcohol was associated with a 3.5-fold higher rate of wound complications compared to chlorhexidine–alcohol [19]. However, a randomized trial comparing iodine povacrylex and chlorhexidine gluconate for skin antisepsis in extremity fractures found fewer SSIs with iodine povacrylex in closed fractures, while outcomes were similar for open fractures [20]. Surgeons must weigh factors such as cost and potential adverse effects, like allergies, when selecting an antiseptic for skin preparation.

The technique of skin closure and postoperative wound management is another critical factor in preventing SSIs. However, evidence comparing closure methods is limited. A meta-analysis evaluating SSIs after closure with staples versus sutures in elective knee and hip surgeries found a higher SSI risk with staples [21]. These findings may be relevant to ACLR, which typically involves two to three puncture wounds with additional small incisions, depending on the graft used—quadriceps tendon (QT), bone–patellar tendon–bone (BPTB), or hamstring tendon (HT)—and any concomitant procedures such as osteotomy, meniscal surgery, or extra-articular tenodesis [21,22].

## 4. Graft Selection and Preparation

### 4.1. Graft Selection

Graft selection in ACLR is a critical yet controversial topic, and it is one of the factors affecting graft rupture, reoperation, and infection rates [23,24]. Historically, allografts were thought to be more predisposed to postoperative knee infections compared to autografts [5]. Additionally, although the incidence of infectious disease transmission via allografts is nearly negligible, the rare possibility of viral transmission, such as hepatitis and HIV, remains an important consideration [25]. However, utilizing novel techniques for allografts that include low-dose irradiation and standardized chemical protocols for sterilization, and utilizing the technique of presoaking the graft in vancomycin, the most recent research indicates that autografts and allografts demonstrate comparable rates of postoperative infection [26]. Several studies have demonstrated that there is no increased infection risk associated with the use of allograft tissue compared to autografts in primary ACLR [27,28]. Therefore, allografts are a safe option when processed appropriately for ACLR and may not carry the burden of increased infection risk. Given the recent evidence suggesting that autografts and allografts are equivalent in terms of infection risk, decisions on graft type are frequently dictated by patient characteristics and surgeon preference rather than infection risk [29].

Among autografts, the HT has been found to be a greater risk factor for infection after ACLR than the QT and BPTB [30]. Numerous studies have indicated a higher infection risk associated with HT grafts when compared to BPTB grafts [24,31,32]. HT autografts exhibited a higher contamination rate during autograft preparation and harvest than BPTB autografts (13% vs. 10%, respectively), which may lead to the increase in postoperative infection following ACLR [33]. Meta-analyses have demonstrated that HT autografts display a higher susceptibility to postoperative infections, with reported infection rates of 1.1% for HT compared to 0.7% for BPTB autografts [26,34,35]. Also, further research has demonstrated a significantly higher risk of infection using HT autografts when compared to QT autografts for ACLR [32,36]. Although the cause of the increased risk of infection associated with HT autografts is still debated, it is widely recognized that HT autografts often require an extended preparation time and cannulated harvesting instruments. Additionally, it has been postulated that the increase in infection rates may be due to the hamstring harvest site’s location being in close proximity to the tibial tunnel [32,34,37].

### 4.2. Graft Preparation

Perioperative intravenous antibiotic prophylaxis may significantly reduce the postoperative infection rate after ACLR, although intravenous (IV) administration does not achieve tissue levels above the minimum inhibitory concentration (MIC) in tendons and joints due to vascular supply limitations [38]. Recently, the topical application of vancomycin on the graft during ACLR has increased in clinical practice, and numerous series have demonstrated a significant reduction in the incidence of postoperative knee joint infections [39,40,41,42]. For instance, one technique described involves soaking the ACL graft in a surgical sponge saturated with 5 mg/mL vancomycin solution for 15 min [40]. Studies have shown that up to 14% of hamstring ACL grafts are contaminated with bacteria after harvesting and preparation [43]. Topical soaking of vancomycin has been proven to safely eradicate this contamination without any negative effects on the biomechanical properties of the grafts [44,45]. Furthermore, there is no reported evidence of chondrotoxic or osteotoxic concentrations within the knee joint after soaking the ACL graft in vancomycin [46]. Various meta-analyses, involving over 10,000 patients, have confirmed the efficacy and safety of topical vancomycin application on the graft during ACLR, with postoperative infection rates as low as 0.09% [47,48]. Despite the lack of randomized control trials (RCTs), vancomycin presoaking is widely used among surgeons performing ACLR and is considered a highly effective strategy for preventing postoperative infections. However, the evidence supporting its efficacy remains limited to retrospective and observational studies [39,40,41,42]. Variability in soaking protocols, vancomycin concentrations, and outcome reporting makes comparisons between studies heterogeneous and challenging [39,41]. Future RCTs are necessary to standardize the method and practice of vancomycin presoaking and therefore strengthen the efficacy of its use in infection prevention.

## 5. Diagnosis

Early diagnosis of infection following ACLR is crucial for successful treatment and preservation of the graft. Patients may present with a variety of symptoms, including general malaise, loss of appetite, fever, chills, pain, redness, limited mobility, and impaired function of the affected knee joint. The most sensitive findings include pain with joint movement, limited mobility, and joint swelling or effusion within 24 h to 6 weeks post-reconstruction, typically after a period of symptom relief [49,50]. Joint aspiration and venous blood sampling are fundamental for diagnostic and appropriate therapeutic planning. Elevated C-reactive protein (CRP) and leukocyte counts are key indicators for joint aspiration. Turbid or flaky synovial fluid with a cell count > 20,000/µL suggests a knee joint empyema. A cell count > 50,000/µL, in the absence of crystals, is essentially diagnostic for septic arthritis. Also, a neutrophil granulocyte ratio > 90% yields a sensitivity of 94% and specificity of 100% for septic arthritis. Neutrophil granulocyte ratio values > 70% are a clear indication of a bacterial infection of the joint [32]. Procalcitonin (PCT) levels may also provide guidance in cases of borderline synovial analysis results [51]. During aspiration, aerobic and anaerobic blood culture bottles should be inoculated with synovial fluid and incubated for at least 14 days. Since microbiological detection of the causative pathogen is successful in less than 75% of infections following ACLR, these samples are often limited in their use for acute diagnosis. However, they remain valuable for adjusting antimicrobial therapy when a pathogen is identified [52].

Advanced imaging may be useful in the diagnosis of infection following ACLR. Magnetic resonance imaging (MRI) findings of effusion, synovial thickening, contrast enhancement within the synovium, and cystic changes within the ACL graft tunnels may indicate a postoperative infection [53]. However, given timing and cost considerations, an MRI may delay the diagnosis and treatment of infection after ACLR, and may be obsolete if a knee joint infection is clinically suspected. However, an MRI or other advanced imaging may be useful in cases of infection persistence despite repeated arthroscopic irrigations and adequate antibiotic treatment in cases of infection after ACLR. In these presentations, an MRI is typically recommended to exclude periarticular abscesses, infected periarticular or perimeniscal cysts, and bone involvement or osteomyelitis [53]. Plain radiographs of the knee joint are also used in standard diagnostic methods when there is clinical suspicion for postoperative knee joint infection and may help to assess possible bony involvement [53].

## 6. Microbiology

Although isolating the causative pathogen of infection may not always be possible with culture data, there are certain pathogens that are more commonly associated with postoperative ACLR infection [54]. A recent systematic review compiling data from 33 studies on pathogens cultured from postoperative ACLR infections reported a total of 508 pathogens from 27 different species [3]. The most frequently detected pathogens are Staphylococcus species, particularly Staphylococcus aureus and coagulase-negative staphylococci (CoNS), such as Staphylococcus epidermidis [3,55,56]. These organisms are responsible for up to 90% of post-ACLR infections, with CoNS accounting for nearly half of these cases [57]. Within the CoNS group, *S. epidermidis* is the most frequently detected species, accounting for 36% of methicillin-sensitive cases and 29% of methicillin-resistant cases [3]. Other species within the CoNS group include *S. caprae*, *S. capitis*, *S. lugdunensis*, *S. hominis*, *S. haemolyticus*, *S. schleiferi*, and *S. warneri* [3,58].

Additional pathogens observed in post-ACLR infections include Enterobacter species, Enterococcus faecalis, and Cutibacterium acnes (formerly known as Propionibacterium acnes) [3,55]. Notably, *C. acnes* is particularly associated with delayed-onset infections, especially in cases involving bioabsorbable screws [58]. The anaerobic nature of *C. acnes*, combined with its ability to form biofilms, significantly complicates treatment, as biofilms protect the pathogens from both antibiotics and host immune responses [5,58]. Diagnosis of *C. acnes* infections is often delayed due to the organism’s slow growth, with cultures typically requiring extended incubation periods of up to two weeks [5,57]. The propensity of these bacteria to form biofilms on graft and suture materials poses a significant challenge in the management and eradication of infection [5,58]. Biofilms, irrespective of the causative organism, may contribute to culture-negative infections, which have been reported in 15–30% of cases following ACLR [5,57]. Further advancements in pathogen detection may benefit from integrating molecular diagnostics, such as polymerase chain reaction (PCR) or next-generation sequencing, into clinical practice. These techniques have the potential to enhance the detection of difficult-to-culture pathogens and identify organisms contributing to biofilm formation [5,55]. Expanding the use of molecular diagnostics in studies on ACLR infections may improve the understanding of pathogen profiles and create more effective treatment strategies.

Infections may also be caused by Gram-negative bacteria such as *Pseudomonas aeruginosa*, Enterobacter species, and occasionally *Escherichia coli* [5]. While these pathogens are less common, they are often associated with a more aggressive disease course and may require broad-spectrum antibiotic therapy [5,58,59]. Additionally, polymicrobial infections, although uncommon, are difficult to manage and typically require a combination of surgical debridement along with a prolonged course of antibiotic regimens [55,60].

Fungal infections following ACLR are rare but do present a significant problem due to their gradual progression and potential for significant joint destruction. Fungal infections after ACLR are more commonly found in immunocompromised patients, but have also been reported in immunocompetent individuals [55,61]. Reported cases of infection after ACLR have identified pathogens such as *Candida albicans*, Aspergillus species, and *Rhizopus microsporus* (a cause of mucormycosis), as well as other species from Mucorales [55,58,61,62,63,64].

Fungal infections after ACLR often present with a delayed onset and are not typically detected on standard cultures, thus making diagnosis challenging [55,63]. Advanced diagnostic techniques, such as polymerase chain reaction (PCR) or mass spectrometry, may be necessary to confirm the presence of fungal pathogens [5]. Fungal infections require a high index of suspicion, as they can progress to devastating outcomes, including cartilage loss or degeneration, bony destruction, and osteolysis, if the infection is left untreated [64]. Early detection, combined with prompt removal of implants and grafts, along with aggressive antifungal therapy and debridement, is crucial for successful management in fungal infections after ACLR [55].

Patients with underlying immunosuppressive conditions, such as diabetes, chronic corticosteroid use, or autoimmune diseases, are at a significantly higher risk of developing infections following ACLR [5,64]. These patients are more susceptible to atypical pathogens, including anaerobes and fungi, which may complicate standard treatment protocols [58,60]. Infections in immunocompromised patients often have an atypical clinical presentation, with less pronounced systemic symptoms, making early diagnosis more challenging [5,55]. Although rare, infection caused by Clostridioides difficile has been documented in immunosuppressed patients following ACLR, emphasizing the need for heightened clinical awareness in this patient population [64,65]. Management in these patients requires a more aggressive approach, often combining prolonged, broad-spectrum antibiotic therapy with close monitoring to prevent rapid disease progression and complications or adverse outcomes [55,64].

## 7. Antibiotic Management

A critical aspect of reducing the risk of infections after ACLR is the strategic use of antibiotics during the perioperative period. This includes not only preoperative prophylaxis prior to ACLR but also careful consideration of postoperative antibiotic protocols when managing infection after ACLR. Optimizing the timing, dosage, and selection of antibiotics is critical for minimizing the risk of postoperative infections. Such measures can prevent severe complications, including cartilage degeneration, loss of function, and graft failure, thereby improving patient outcomes following ACLR [30,57,66].

### 7.1. Preoperative Antibiotic Prophylaxis

Preoperative antibiotic prophylaxis plays a crucial role in reducing infection risk [38,67]. The standard practice includes administering a single dose of a cephalosporin, typically cefazolin (2 g) or cefuroxime (1.5 g), 30 to 60 min prior to surgical incision [57]. For patients with a type 1 allergy or anaphylaxis to penicillins or cephalosporins, or a history of MRSA colonization, vancomycin (1 g or 15 mg/kg, up to a maximum of 2500 mg) is recommended as an alternative [30,57].

Extending antibiotic prophylaxis beyond 24 h postoperatively is discouraged, as it has been linked to increased infection rates, particularly in primary knee arthroplasties [68]. Additionally, the use of oral antibiotics for postoperative prophylaxis is generally unnecessary and may lead to side effects, including reduced bioavailability and diminished efficacy [5].

### 7.2. Empirical Antibiotic Treatment Prior to Culture Results

When an infection is suspected following ACLR, prompt intervention is essential. Empirical antibiotic therapy should begin immediately after joint aspiration in acute cases, prior to waiting for culture results [55,57]. This initial treatment generally includes a combination of a beta-lactam/beta-lactamase inhibitor, such as ampicillin/sulbactam (3 × 3 g i.v.) or amoxicillin/clavulanic acid (4 × 2.2 g i.v.), along with vancomycin (dosed to maintain trough levels of 15–20 mg/L) or daptomycin (1 × 8–10 mg/kg i.v.) to cover both Gram-positive and Gram-negative bacteria [30,57]. For patients without an anaphylactic penicillin allergy, a first- or second-generation cephalosporin, such as cefazolin (3 × 2 g i.v.) or cefuroxime (3 × 1.5 g i.v.), is a suitable alternative [57]. In cases of severe (type 1) allergies to beta-lactams, daptomycin (1 × 8–10 mg/kg) is preferred [57].

It is important to delay antibiotic treatment until after synovial fluid samples are collected, if clinically possible, as early administration may compromise microbiological results and lead to false negatives [30,57]. Additionally, combining arthroscopic debridement with antibiotic therapy is recommended to reduce bacterial load and excise infected tissue [55,57].

### 7.3. Postoperative Antibiotic Therapy Based on Culture Results

After culture results are obtained, the antibiotic regimen should be tailored to the identified pathogens and their susceptibility profiles [57,64]. This targeted approach enhances treatment efficacy while reducing the risk of antibiotic resistance [64]. For cases involving implants, biofilm-active antibiotics like rifampin (2 × 450 mg) are recommended, given the tendency of implant materials to support biofilm formation, which complicates eradication [57,69]. Previous studies have supported the use of oral rifampin in combination with levofloxacin for staphylococcal infection after ACLR with retained grafts [60].

Culture-negative infections are particularly challenging and require broad-spectrum intravenous antibiotic therapy to cover the most common pathogens. A commonly used regimen includes ampicillin/sulbactam (3 × 3 g i.v.) for two weeks, followed by a biofilm-active combination of rifampin and levofloxacin (2 × 450 mg and 2 × 500 mg, respectively) [57].

### 7.4. Duration of Antibiotic Course

The optimal duration of antibiotic therapy for infections following ACLR remains a topic of debate. Current protocols generally recommend a combination of intravenous (IV) and oral antibiotics, with IV therapy lasting one to two weeks, followed by four to five weeks of oral antibiotics [57]. The decision to transition from IV to oral therapy is typically guided by clinical response, normalization of inflammatory markers such as CRP, and the resolution of local symptoms [30,57].

In cases involving biofilm-forming organisms or when grafts and fixation devices are retained, a longer antibiotic course—up to 12 weeks—may be necessary [24]. Collaboration with infectious disease specialists is crucial to determine the appropriate duration and choice of antibiotic therapy, especially in complex infections involving avascular tissues or implants. The primary objective is to fully eradicate the infection while preserving knee joint integrity and achieving optimal functional outcomes [55]. Future research should focus on optimizing the duration and combination of antibiotics and evaluating the role of biofilm-active agents. RCTs may assist in refining antibiotic protocols and thus further validating their efficacy in the prevention and management of infections after ACLR.

## 8. Therapeutic Management

Management of infection after ACLR is a complex challenge requiring careful consideration of both patient-specific and surgical factors. Timely diagnosis is critical when infection is suspected, as postoperative infection can significantly compromise outcomes by leading to knee dysfunction, cartilage damage, arthrofibrosis, and potential graft failure [57,66,70].

Upon confirming infection after ACLR, the standard approach involves using culture-specific antibiotics in conjunction with surgical irrigation, debridement, and graft inspection [71]. Typically, surgeons opt to retain the ACL graft [71], although no definitive guidelines exist for graft retention versus removal, leaving this decision largely based on the surgeon’s clinical judgment [55]. There are generally three approaches: graft retention with arthroscopic debridement (sometimes multiple rounds), graft removal following unsuccessful debridement, or immediate graft removal [55].

Furthermore, there has been much work carried out to investigate low-grade infection or subclinical infection with bacterial burden on ACLR grafts and its impact in subsequent ACLR failure and effect of revision ACLR situations [72]. These low-grade infections with bacterial biofilms negatively impact the collagen fibers of ACLR grafts, therefore decreasing their strength to failure and therefore increasing the risk of ACLR failure [72]. Additionally, research has demonstrated tibial tunnel widening is associated with increased bacterial DNA, again highlighting the effect of bacterial burden on ACLR, even in subclinical settings [72,73]. Further research may need to investigate causal relationship between bacterial burden and its impact on ACLR failure, and further define these potential subclinical low-grade infections in knees after ACLR. Current management strategies for the treatment of infection after ACLR mainly focus on overt infections. However, the possibility of these low-grade or subclinical infections highlights the need for improved understanding of the relationship between bacterial burden and ACLR failure. Further research is needed to determine appropriate diagnostic methods and management strategies for subclinical or low-grade infections.

### 8.1. Graft Retention with Repeated Arthroscopic Debridement

The prevailing consensus in the literature favors antibiotic therapy combined with surgical debridement while retaining the ACL graft [8,64,70,71]. Studies indicate higher failure rates when relying solely on antibiotics without surgical intervention [5,66]. Therefore, initial management of suspected septic arthritis typically involves prompt surgical debridement [5], followed by culture-specific antibiotics administered for multiple weeks, potentially transitioning from intravenous to oral antibiotic therapy [55,66]. Evidence suggests that surgical debridement with graft retention achieves over 80% success rates, leading to better postoperative function and reduced morbidity compared to graft removal followed by delayed revision ACLR [5,30,74]. However, if intraoperative findings indicate issues such as graft degeneration, fixation failure, or tears, graft removal may be warranted. Surgeons may have a low threshold for graft removal if there are concerns about the ACL graft during the initial debridement [75,76].

### 8.2. Graft Removal After Failed Debridement

Multiple debridements, i.e., three or more, have been associated with a higher likelihood of requiring graft removal [24]. However, this association is debatable, as even after multiple debridements, surgeons may opt to retain a stable ACL graft to prevent its loss. Multiple debridements alone do not necessitate graft removal. Individualized treatment is essential, with graft evaluation and a review of reconstruction procedures guiding the decision between graft removal and retention. In cases where the infection extends to the bone, causing osteomyelitis and significant graft damage, acute graft removal may be necessary [55,64]. Indications for graft removal after repeated debridement include poor graft integrity upon probing or evidence of purulent exudate covering the graft [66]. Special cases, such as fungal or tubercular infections, may require extended antimicrobial treatment and potentially open arthrotomy instead of arthroscopy due to delayed diagnosis and treatment challenges [66].

### 8.3. Immediate Graft Removal Versus Graft Retention

Though less commonly employed, immediate graft removal may be favored in specific scenarios [76]. There is conflicting evidence regarding graft removal versus retention and the optimal timeframe for removing an ACLR graft [75]. For example, some surgeons report a higher rate of graft removal if a delayed diagnosis of septic arthritis is given beyond seven days after ACLR [66]. These authors also noted that prolonged antibiotic therapy course duration increased the likelihood of graft removal [66]. However, this timeframe of delayed diagnosis beyond seven days can be challenging to interpret, as many infections may not manifest until weeks or even months after ACLR. Immediate removal has also been recommended when the graft is deemed nonfunctional due to elongation or is found coated with thick purulent exudate upon arthroscopic evaluation [66]. The majority of the existing literature supports graft retention following appropriate debridement and antibiotic therapy [76]. However, graft retention may result in additional surgeries, increased costs, and prolonged hospitalization [76]. Moreover, persistent infection with graft retention can lead to cartilage damage, arthrofibrosis, and subsequent graft failure [76].

There is no true consensus on which clinical scenarios are best treated with immediate graft removal. There is a delicate balance in the decision between immediate removal and repeat debridements to maximize long-term outcomes for patients. Therefore, the senior author’s preference is retention of the ACL graft whenever possible.

### 8.4. Timing of Revision ACLR

The timing of revision ACLR following graft removal for infection varies widely, ranging from six weeks to nine months [30,66]. The European Society of Sports Traumatology, Knee Surgery, and Arthroscopy (ESSKA) and European Bone and Joint Infection Society (EBJIS) guidelines recommend waiting three to six months for revision if osteomyelitis is present in the femur or tibia [30]. In cases without bone involvement, reconstruction may be considered as early as six weeks if the clinical picture improves, CRP levels decrease, and the infection is adequately controlled [30]. Graft selection during revision should consider factors that may predispose to recurrent infection with the goal of limiting factors that may lead to recurrent infection.

## 9. Conclusions

Postoperative infection following ACLR is a rare but serious complication that requires prompt diagnosis and rigorous management. Prevention strategies, including meticulous skin preparation, antibiotic prophylaxis, and graft presoaking in vancomycin, have demonstrated significant reductions in infection rates. Early intervention with appropriate surgical debridement and culture-specific antibiotics is essential for preserving graft integrity and knee function. While graft retention is often preferred, individualized treatment remains crucial, especially in complex cases involving graft damage or osteomyelitis. Continued research is needed to refine management protocols and optimize long-term outcomes for patients experiencing infections after ACLR.

## Figures and Tables

**Table 1:** Key findings of the review.

Postoperative infections after ACLR occur in 0.1% to 2% of cases and can severely affect recovery, leading to graft failure, knee dysfunction, and prolonged treatment.
Timely diagnosis and management of infections are critical to avoid complications such as arthrofibrosis, cartilage damage, and reduced knee function.
Effective prevention includes preoperative skin preparation, antibiotic prophylaxis, and maintaining sterile surgical techniques.
No significant difference in infection rates between autografts and allografts when modern sterilization techniques are applied.
Hamstring tendon autografts have a higher infection risk than bone–patellar tendon–bone and quadriceps tendon autografts.
Soaking ACL grafts in vancomycin has shown infection rates as low as 0.09%.
Prompt recognition of symptoms like fever, joint swelling, and limited mobility is critical for diagnosing infections early and preventing severe outcomes.
Staphylococcus species, particularly Staphylococcus aureus and coagulase-negative staphylococci (CoNS), are the most common pathogens in post-ACLR infections.
Immediate initiation of empirical antibiotic therapy after joint aspiration is recommended, with a focus on culture-specific adjustments once results are available.
Graft retention combined with arthroscopic debridement and antibiotic therapy achieves high success rates and is often preferred, but individualized decisions are necessary based on graft integrity.
Further studies are needed to address low-grade infections, bacterial biofilms, and their contribution to graft failure, as well as to refine guidelines for infection management in ACLR.

## Data Availability

Data are contained within the article.

## Abbreviations

ACL: anterior cruciate ligament; ACLR, anterior cruciate ligament reconstruction; AORN, Association of periOperative Registered Nurses; BPTB, bone–patellar tendon–bone; CDC, Centers for Disease Control and Prevention; CoNS, coagulase-negative staphylococci; CRP, C-reactive protein; EBJIS, European Bone and Joint Infection Society; ESSKA, European Society of Sports Traumatology, Knee Surgery, and Arthroscopy; HT, hamstring tendon; IV, intravenous; MIC, minimum inhibitory concentration; MRI, magnetic resonance imaging; MRSA, methicillin-resistant Staphylococcus aureus; PCR, polymerase chain reaction; PCT, procalcitonin; QT, quadriceps tendon; RCT, randomized controlled trial; SSI, surgical site infection.
